# Supplementary material for: Analysis of corneal real astigmatism and high order aberration changes that cause visual disturbances after lower eyelid epiblepharon repair surgery
Source: Sci Rep. 2020 May 4;10:7498. doi: 10.1038/s41598-020-64386-6 (PMC7198593; doi:10.1038/s41598-020-64386-6)
Supplement: Supplementary file 1 — Supplementary Information. [file 41598_2020_64386_MOESM1_ESM.docx]

**Analysis of corneal real astigmatism and high order aberration changes that cause visual disturbances after lower eyelid epiblepharon repair surgery**

Dong Cheol Lee

**Supplementary Figures**


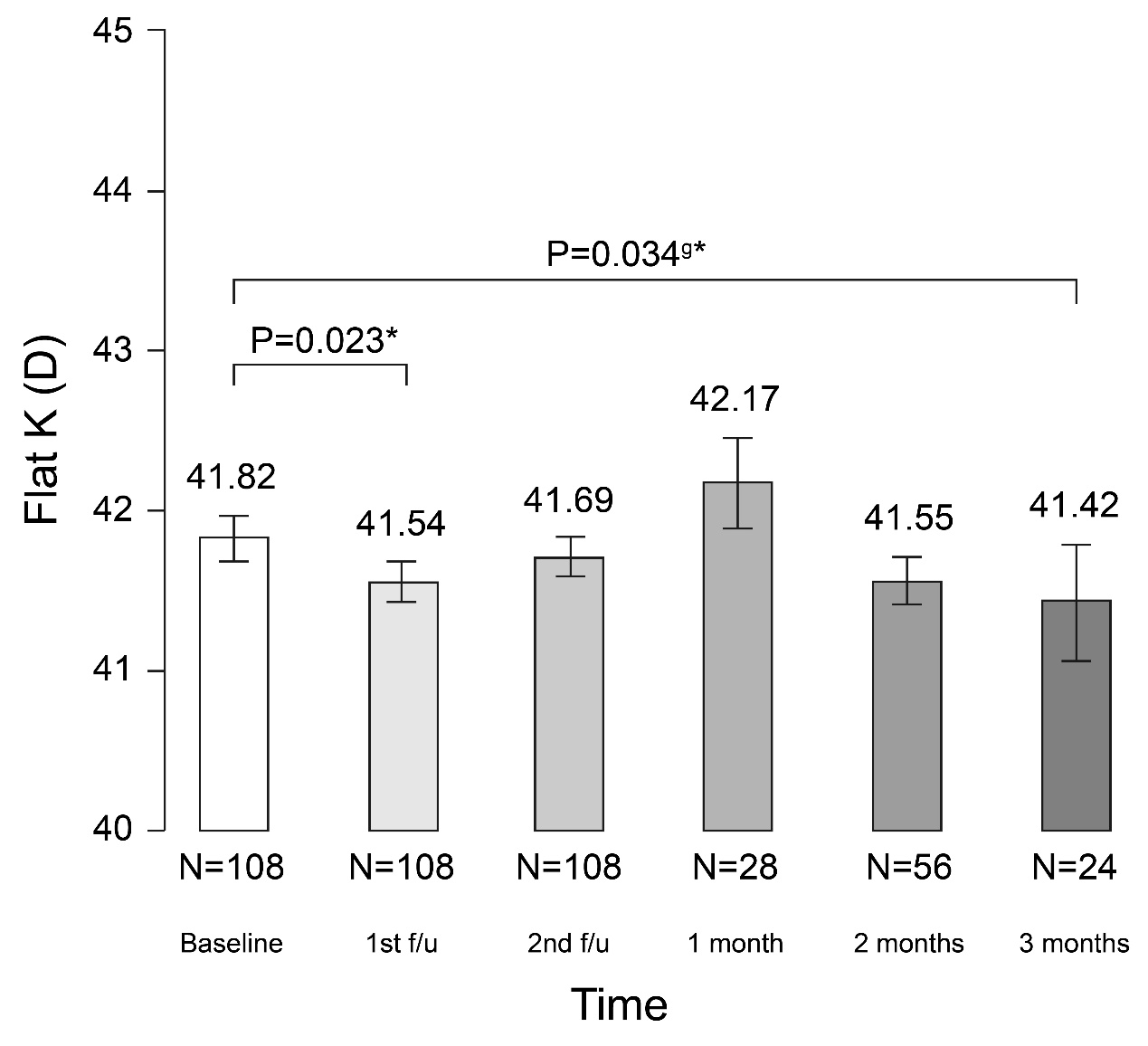


**Fig. S1** Change in flat K according to follow-up (f/u) time expressed as mean ± standard error

Wilcoxon signed-rank test^g^. *p* value* < 0.05, *p* value** < 0.01





**Fig. S2** Change in mean K according to follow-up (f/u) time expressed as mean ± standard error

*p* value* < 0.05, *p* value** < 0.01





**Fig. S3** Change in astigmatism according to follow-up (f/u) time expressed as mean ± standard error

Wilcoxon signed-rank test^g^. *p* value* < 0.05, *p* value** < 0.01





**Fig. S4** Change in sphere according to follow-up (f/u) time expressed as mean ± standard error

Wilcoxon signed-rank test^g^. *p* value* < 0.05, *p* value** < 0.01





**Fig. S5** Change in axis according to follow-up (f/u) time expressed as mean ± standard error

Wilcoxon signed-rank test^g^. *p* value* < 0.05, *p* value** < 0.01





**Fig. S6** Change in steep K according to follow-up (f/u) time expressed as mean ± standard error





**Fig. S7** Change in spherical equivalent (SE) according to follow-up (f/u) time expressed as mean ± standard error





**Fig. S8** Change in cylinder according to follow-up (f/u) time expressed as mean ± standard error





**Fig. S9** Change in coma according to follow-up (f/u) time expressed as mean ± standard error

*p* value* < 0.05, *p* value** < 0.01





**Fig. S10** Change in trefoil according to follow-up (f/u) time expressed as mean ± standard error

Wilcoxon signed-rank test^g^. *p* value* < 0.05, *p* value** < 0.01





**Fig. S11** Change in spherical aberration (SA) according to follow-up (f/u) time expressed as mean ± standard error

Wilcoxon signed-rank test^g^. *p* value* < 0.05, *p* value** < 0.01





**Fig. S12** Change in total root mean square (RMS) (D) according to follow-up (f/u) time expressed as mean ± standard error





**Fig. S13** Change in total root mean square (RMS) (µm) according to follow-up (f/u) time expressed as mean ± standard error





**Fig. S14** Change in defocus according to follow-up (f/u) time expressed as mean ± standard error





**Fig. S15** Change in 2^’^ astigmatism according to follow-up (f/u) time expressed as mean ± standard error
